# Differences in conformational dynamics within the Hsp90 chaperone family reveal mechanistic insights

**DOI:** 10.3389/fmolb.2014.00004

**Published:** 2014-06-10

**Authors:** Christian Graf, Chung-Tien Lee, L. Eva Meier-Andrejszki, Minh T. N. Nguyen, Matthias P. Mayer

**Affiliations:** Ruprecht-Karls-Universität Heidelberg, Zentrum für Molekulare Biologie der Universität HeidelbergHeidelberg, Germany

**Keywords:** hydrogen exchange mass spectrometry, chaperones, Hsp90, dynamics, conformation

## Abstract

The molecular chaperones of the Hsp90 family are essential in all eukaryotic cells. They assist late folding steps and maturation of many different proteins, called clients, that are not related in sequence or structure. Hsp90 interaction with its clients appears to be coupled to a series of conformational changes. Using hydrogen exchange mass spectrometry (HX-MS) we investigated the structural dynamics of human Hsp90β (hHsp90) and yeast Hsp82 (yHsp82). We found that eukaryotic Hsp90s are much more flexible than the previously studied *Escherichia coli* homolog (EcHtpG) and that nucleotides induce much smaller changes. More stable conformations in yHsp82 are obtained in presence of co-chaperones. The tetratricopeptide repeat (TPR) domain protein Cpr6 causes a different amide proton protection pattern in yHsp82 than the previously studied TPR-domain protein Sti1. In the simultaneous presence of Sti1 and Cpr6, protection levels are observed that are intermediate between the Sti1 and the Cpr6 induced changes. Surprisingly, no bimodal distributions of the isotope peaks are detected, suggesting that both co-chaperones affect both protomers of the Hsp90 dimer in a similar way. The cochaperones Sba1 was found previously in the crystal structure bound to the ATP hydrolysis-competent conformation of Hsp90, which did not allow to distinguish the mode of Sba1-mediated inhibition of Hsp90's ATPase activity by stabilizing the pre- or post-hydrolysis step. Our HX-MS experiments now show that Sba1 binding leads to a protection of the ATP binding lid, suggesting that it inhibits Hsp90's ATPase activity by slowing down product release. This hypothesis was verified by a single-turnover ATPase assay. Together, our data suggest that there are much smaller energy barriers between conformational states in eukaryotic Hsp90s than in EcHtpG and that co-chaperones are necessary in addition to nucleotides to stabilize defined conformational states.

## Introduction

Hsp90 chaperones control stability and activity of some 200 native or near native protein clients, most of which are regulatory components of signal transduction pathways such as receptors, protein kinases and transcription factors (Pratt and Toft, 1997; Pearl and Prodromou, 2000; Young et al., 2001; Wegele et al., 2004; DeZwaan and Freeman, 2008).Through chaperoning these clients Hsp90s are involved in the regulation of cell homoeostasis, cell cycle, proliferation, differentiation, and programmed cell death.

**Figure 1:**
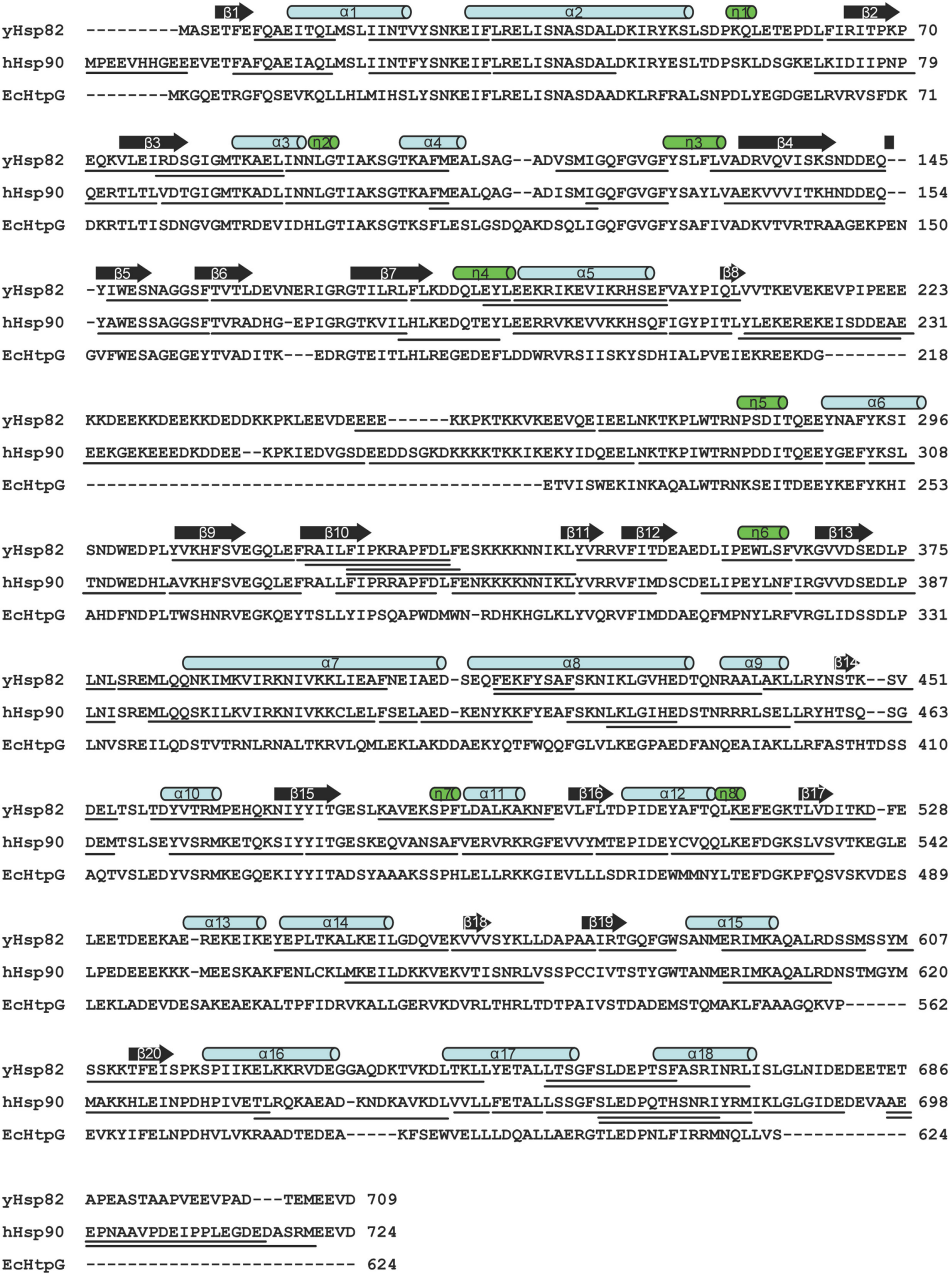
**Comparison of human, yeast and *E. coli* Hsp90 proteins**. Clustal W sequence alignment of human, yeast and *E. coli* Hsp90. Secondary structure elements, α helices (α1–18), 3_10_ helices (η1–8), and β strands (β1–20) according to the crystal structure of yHsp82 (PDB ID code 2CG9) are indicated above the alignment. The lines under yHsp82 and hHsp90 sequences indicate the peptic fragments that were analyzed in this study.

**Figure 2:**
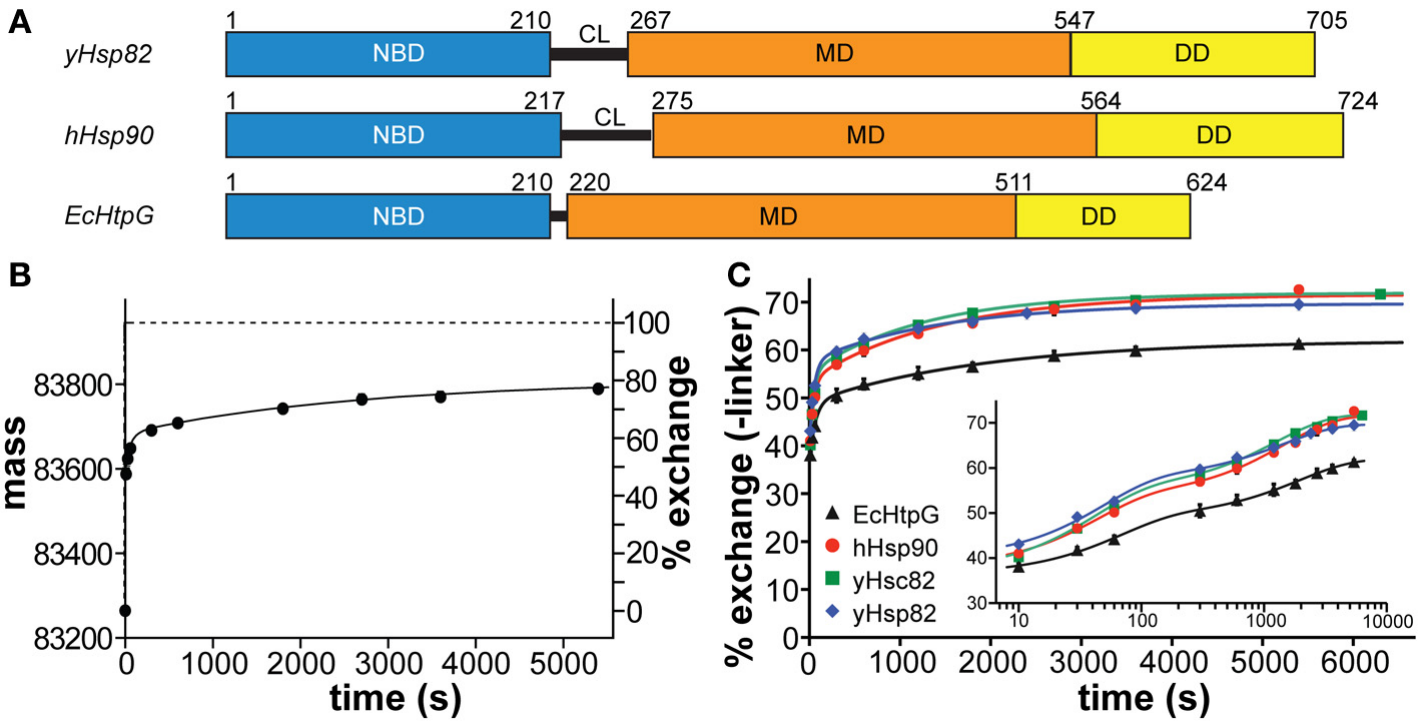
**Comparison of human, yeast and *E. coli* Hsp90 proteins. (A)** Domain structure of human Hsp90β (hHsp90), *S. cerevisiae* Hsp82 (yHsp82) and *E. coli* HtpG (EcHtpG) proteins. NBD, nucleotide binding domain; CL, charged linker; MD, middle domain; DD, dimerization domain. **(B)** HX kinetics of full-length hHsp90. The mass of hHsp90 and the percentage hydrogen exchange, respectively, is plotted vs. the incubation time in D_2_O. The data points are averages of up to four independent experiments and the error bars represent the standard error of mean. The solid line is a fit of a tri-exponential equation [Y = Y_1_·(1-exp(−k_1_·t)) + Y_2_·(1−exp(−k_2_·t)) + Y_3_·(1−exp(−k_3_·t))] to the data points. The dashed line represents the theoretical exchange curve including the back-exchange during the desalting process calculated for the completely unfolded Hsp90 protein using the HXPep program (courtesy of Z. Zhang). **(C)** HX kinetics of full-length hHsp90 (black circles), yHsc82 (green squares), yHsp82 (blue diamonds), and EcHtpG (black triangles; data from (Graf et al., 2009) for comparison). Relative exchange minus the amide hydrogens of the linker [(H_t_–H_linker_)/(H_total_–H_linker_)] is plotted vs. the time in D_2_O. The data points are averages of two to four independent measurements. The error bars represent standard error of mean. The solid lines are the fits of a bi-exponential equation [Y = Y_1_·(1−exp(−k_1_·t)) + Y_2_·(1−exp(−k_2_·t)) + Y_3_] to the data. The inset shows the same data with a logarithmic time scale.

The Hsp90 family of proteins is evolutionary highly conserved with 60% sequence identity between yeast and human and 40% between *E. coli* and human members (Figure 1). In yeast and human two cytosolic Hsp90 proteins with 97 and 86% sequence identity are found, yeast Hsc82 and yeast Hsp82 and human Hsp90α and human Hsp90β, that are expressed at different level and in cell type-specific manner, respectively (Ghaemmaghami et al., 2003; Sreedhar et al., 2004). The homodimeric Hsp90 proteins consist of an N-terminal nucleotide-binding domain (NBD), a middle domain (MD) and a C-terminal dimerization domain (DD) (Figure 2A). Crystal structures of full-length constructs of yeast Hsp82, of *E. coli* HtpG, and of the canine Grp94 show similar domain structures but very different domain orientations, suggesting significant conformational dynamics (Ali et al., 2006; Shiau et al., 2006; Dollins et al., 2007). Based on the structurally related GHKL-ATPases DNA gyrase B and MutL the ATPase cycle of Hsp90 was proposed to involve several conformational changes including ATP-dependent docking of NBD and MD of the same monomer and a dimerization of the two NBDs in the Hsp90 dimer resulting in a twisted intertwined structure (Ali et al., 2006; Pearl and Prodromou, 2006). Several studies using electron microscopy, small angle X-ray scattering (SAXS) and fluorescence resonance energy transfer (FRET) confirmed domain movements in Hsp90 proteins (Shiau et al., 2006; Southworth and Agard, 2008; Hessling et al., 2009; Krukenberg et al., 2009; Mickler et al., 2009). However, it is unclear, whether there is a uniform cycle of conformational changes or whether Hsp90 proteins differ in the basis for their chaperone activity.

A method well suitable to analyze these fundamental concepts of the chaperone mechanism is hydrogen-^1^H/^2^H-exchange (HX) mass spectrometry (MS) because it enables insights into global and local protein stability, secondary structure dynamics and induced alterations in protein conformation (Hoofnagle et al., 2003; Wales and Engen, 2006). We recently analyzed the conformational dynamics of *E. coli* HtpG (EcHtpG) (Graf et al., 2009). We found that HtpG is rather dynamic in the absence of nucleotides and in the presence of ADP. ATP induced a relatively slow stepwise transition to a more tightly folded state, which we called the tensed-state in contrast to the nucleotide-free relaxed state. Here we analyzed three eukaryotic homologs of HtpG, human Hsp90β (hHsp90), yeast Hsc82 (yHsc82), and yeast Hsp82 (yHsp82) to investigate whether the conformational changes detected for HtpG are conserved throughout evolution. We found that yeast and human Hsp90s differ significantly from EcHtpG in respect to rigidity and conformational dynamics and how nucleotides influence conformational dynamics.

## Results

### Deuteron incorporation into human and yeast Hsp90s

To analyze the conformational dynamics of human and yeast Hsp90s we performed HX-MS experiments essentially as described earlier (Rist et al., 2003, 2006; Graf et al., 2009). We found that the yeast and human Hsp90 proteins are extremely flexible at the secondary structure level exchanging in the nucleotide-free state close to 50% of their amide hydrogens within 10 s and almost 80% within 1 h (Figures 2B,C). The exchange kinetics of the eukaryotic Hsp90s contrast the earlier investigated EcHtpG (Graf et al., 2009), which was more stable exchanging only 38% of its amide hydrogens within 10 s and around 60% within 1 h. This difference in mobility is not related to the different length of the charged linker connecting NBD and MD (see Figure 2A) and also apparent when the numbers of linker amide hydrogens are subtracted from the data (Figure 2C). The difference was also not due to the physiological role of the Hsp90 as constitutively high abundant housekeeping or heat shock protein, since we did not detect significant differences between yHsc82 and yHsp82 at this level.

**Figure 3:**
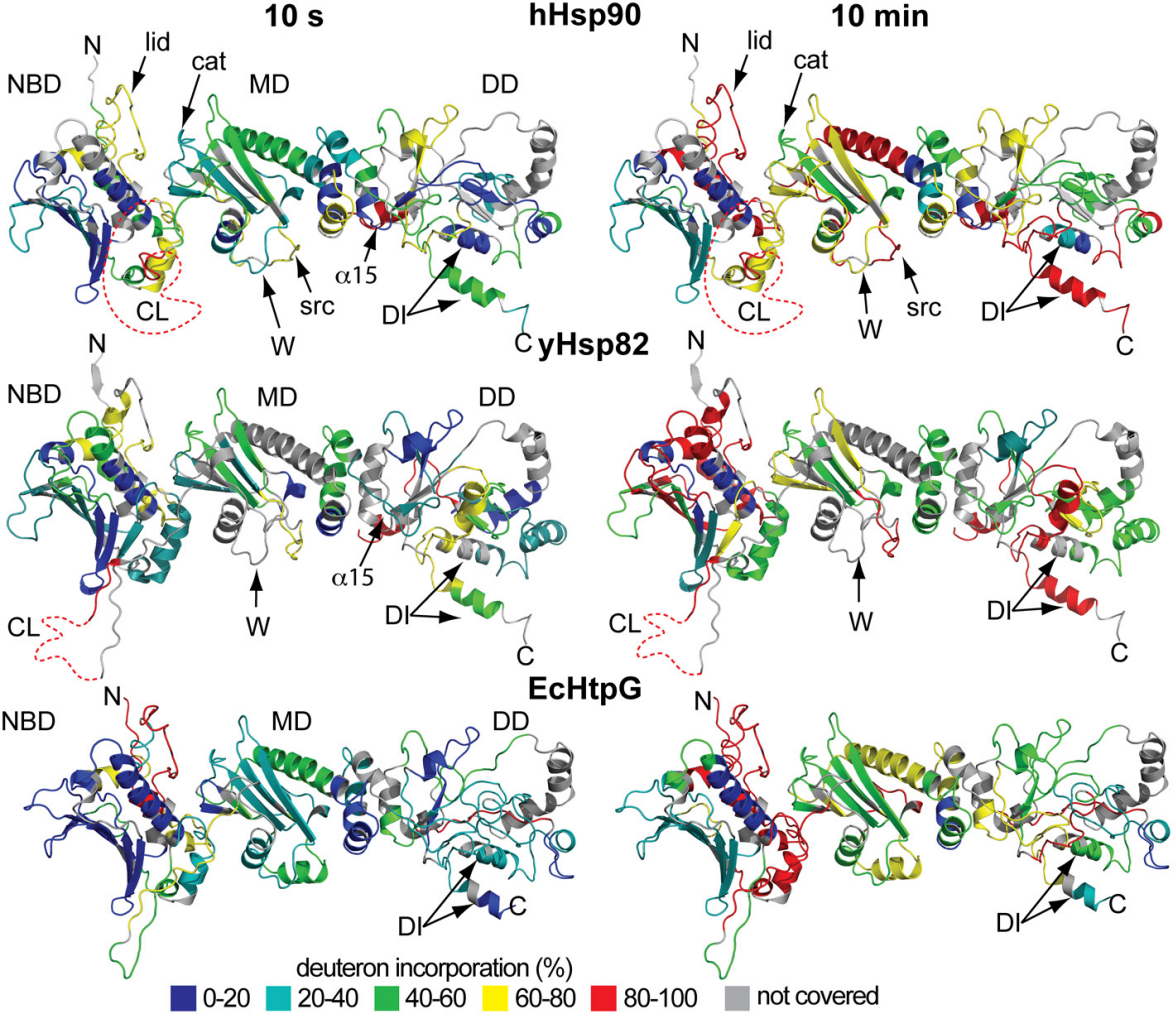
**Localization of fast and slow exchanging regions within the three Hsp90 proteins**. Secondary structure representations of a homology model of hHsp90 onto the structure of yHsp82 [PDB ID code 2CG9; (Arnold et al., 2006; Guex et al., 2009; Kiefer et al., 2009); upper panels], the crystal structure of yHsp82 (PDB ID code 2CG9; middle panels), and a homology model of EcHtpG onto the structure of yHsp82 (PDB ID code 2CG9; lower panels) colored according to the degree of deuteron incorporation after 10 s (left panels) and 10 min (right panels) as indicated. N, N terminus; C, C terminus; NBD, nucleotide binding domain; MD, middle domain; DD, dimerization domain; CL, charged linker; lid, ATP-lid; cat, loop containing the catalytic arginine; DI, dimerization interface. Elements implicated in substrate binding: W, loop containing tryptophan 312 [hHsp90; position 300 in yHsp82; (Sato et al., 2000)]; src, src-loop (Meyer et al., 2003); α15, α helix 15 [numbering according to Figure 1; helix 21 in HtpG; (Harris et al., 2004)]. Structural representation were created in PyMOL (DeLano, W.L. The PyMOL Molecular Graphics System (2002) on World Wide Web http://www.pymol.org).

Online peptic digestion under quench conditions at 0°C allowed us to map fast and slow exchanging regions in the proteins with a sequence coverage of 82, 65, and 69% for the hHsp90, yHsc82 and yHsp82, respectively, including parts of the charged linker, which is not observed in any crystal structure (Figure 1). High secondary structure stability was observed in the NBD except for the ATP-lid [residues 99–133 in hHsp90; homologous to the ATP-lid in GHKL-ATPases (Prodromou et al., 1997a)]. MD and DD are much more flexible than the NBD (Figure 3). Not surprisingly, the charged linker (residues 216–275 in hHsp90), which connects NBD and MD and which is symbolized as dashed line in Figure 3, exchanged almost all of its amide hydrogen within 10 s. A high degree of dynamics was observed in the catalytic loop in the MD (residues 382–402 in hHsp90) and in three structural elements implicated in substrate binding: the loop, which contains tryptophan 312 in hHsp90 [position 300 in yHsp82; (Sato et al., 2000)], the so-called Src-loop [residues 324–340 in yHsp82 and not resolved in the structure; (Meyer et al., 2003)], and α helix 15 (helix 21 in EcHtpG (Harris et al., 2004); Figure 3). Even one or both of the helices involved in the dimer interface incorporated deuterons to a high degree within 10 min consistent with a fast dimer-monomer equilibrium and frequent opening of the DD (Figure 3 right panels) (Richter et al., 2001; Ratzke et al., 2010). Deuteron incorporation in yHsc82 was very similar to yHsp82, indicating that the heat-inducible isoform is not necessarily more stable.

The difference in conformational dynamics between the eukaryotic Hsp90 proteins and EcHtpG were found in many parts of MD and DD. In particular, one of the helices involved in the dimer interface is more stable in EcHtpG than its counterpart in yeast or human Hsp90 suggesting a less dynamic dimer-monomer equilibrium and/or opening of the DD.

### Effects of nucleotides on hydrogen exchange in Hsp90 proteins

**Figure 4:**
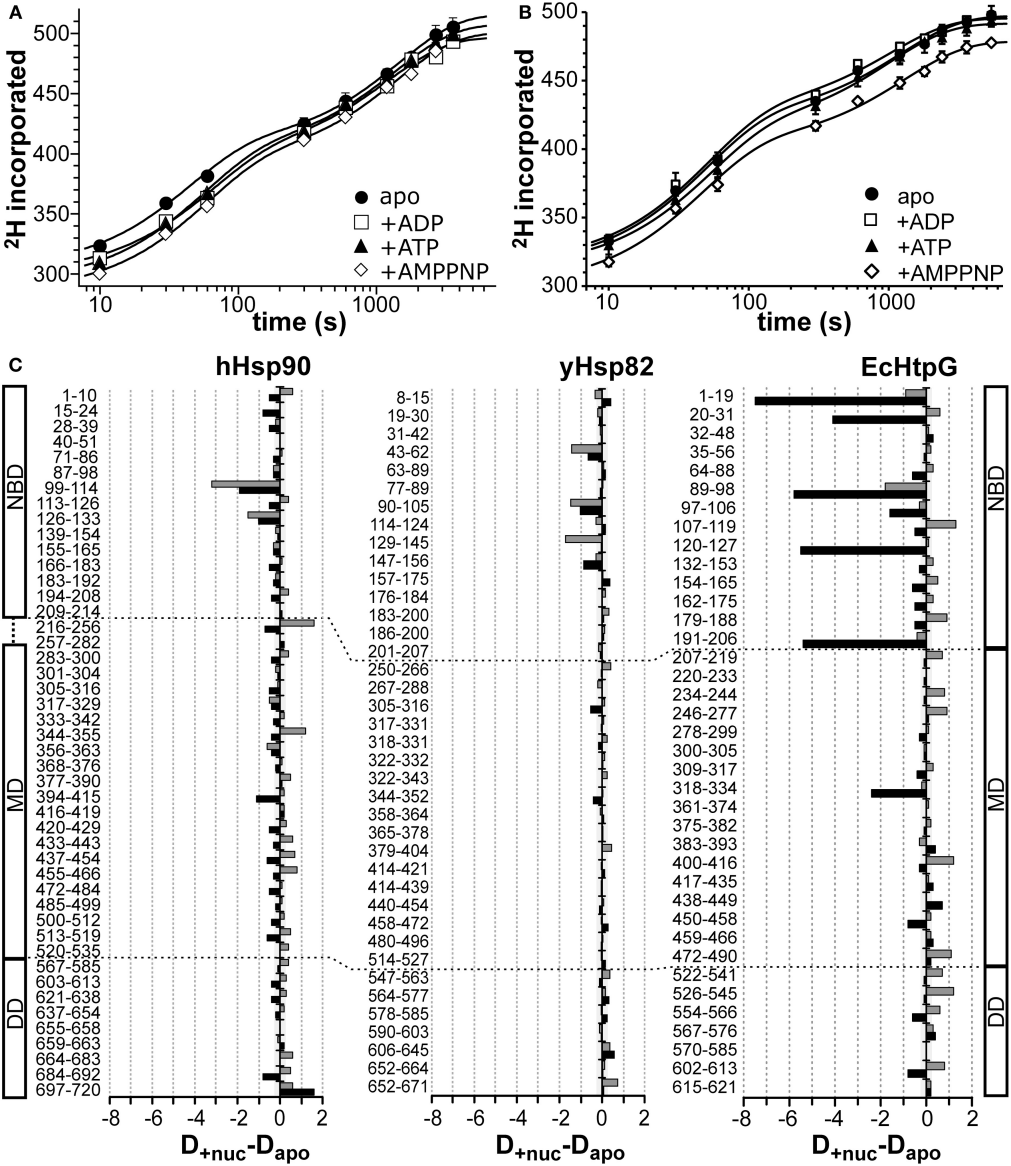
**Nucleotide dependent changes in the HX kinetics of Hsp90 proteins**. Deuteron incorporation into full-length hHsp90 **(A)** and yHsp82 **(B)** in the absence of nucleotides (black circles) and in the presence of ADP (open squares), ATP (black triangles), and AMPPNP (open diamonds). The solid lines are fits of a bi-exponential equation to the data. **(C)** Difference plots of deuterons incorporated in the presence of ATP (black bars) and ADP (gray bars) minus deuterons incorporated in the absence of nucleotides into hHsp90 (left panel), yHsp82 (middle panel) and EcHtpG (right panel, data from Graf et al., 2009) after 30 s in D_2_O. The numbers to the left of each panel indicate the corresponding peptic peptide. Presented are the means of three independent experiments. Domain structure of hHsp90 and EcHtpG are shown left and right of the graph. Dashed lines indicate domain boundaries in the three proteins.

To analyze the influence of nucleotides on the conformational dynamics we pre-incubated the three Hsp90 proteins with a large excess of ADP, ATP, and the non-hydrolyzable ATP analog AMPPNP for at least 10 min before the start of the proton/deuteron exchange reaction. As compared to the previously analyzed EcHtpG, nucleotides only induced small changes in conformational dynamics of the eukaryotic Hsp90 proteins (Figure 4). For hHsp90 we found that all three nucleotides led to a small protection at short time intervals (10, 14, and 23 hydrogens for ADP, ATP and AMPPNP at 10 s) (Figure 4A). For yHsp82 only in the presence of AMPPNP a significant protection was observed in the full-length protein (on average 18 protons) (Figure 4A). These observations are in striking contrast to the 50 protons protection observed in EcHtpG upon addition of ATP (Graf et al., 2009), indicating that in the eukaryotic proteins ATP does not induce a similarly rigid state comparable to the “tensed state” of EcHtpG.

Mapping the nucleotide-induced differences onto the sequence of hHsp90 revealed that a stabilizing effect of ATP was observed in the ATP-lid (residues 99–114 and 126–133; Figure 4C, left panel, black bars). A small stabilization was also observed throughout the entire protein down to the very C-terminus. In particular, the segment following the catalytic arginine 392 exhibited a significant stabilization in the presence of ATP suggesting a contact between the NBD and the MD upon ATP binding consistent with the currently favored ATPase mechanism for Hsp90 proteins. In the presence of ADP, the situation is quite different for hHsp90. Though a protection of the ATP-lid was also observed in the presence of ADP, several segments of significant deprotection occurred throughout the protein contrasting the protection in the presence of ATP (Figure 4C left panel, gray bars). Part of the charged linker (residues 216–256), the src-loop (residues 344–355), and the helical coiled-coil of the MD (residues 433–466) exhibited this deprotection.

For yHsp82 we observed in the presence of ATP a small protection in the NBD and two segments of the MD and a deprotection in the DD (Figure 4C, middle panel, black bars). In the presence of ADP, protection was observed in the NBD and deprotection in parts of the MD and the DD. In particular, the segment encompassing the dimerization interface was deprotected in the presence of ADP.

**Figure 5:**
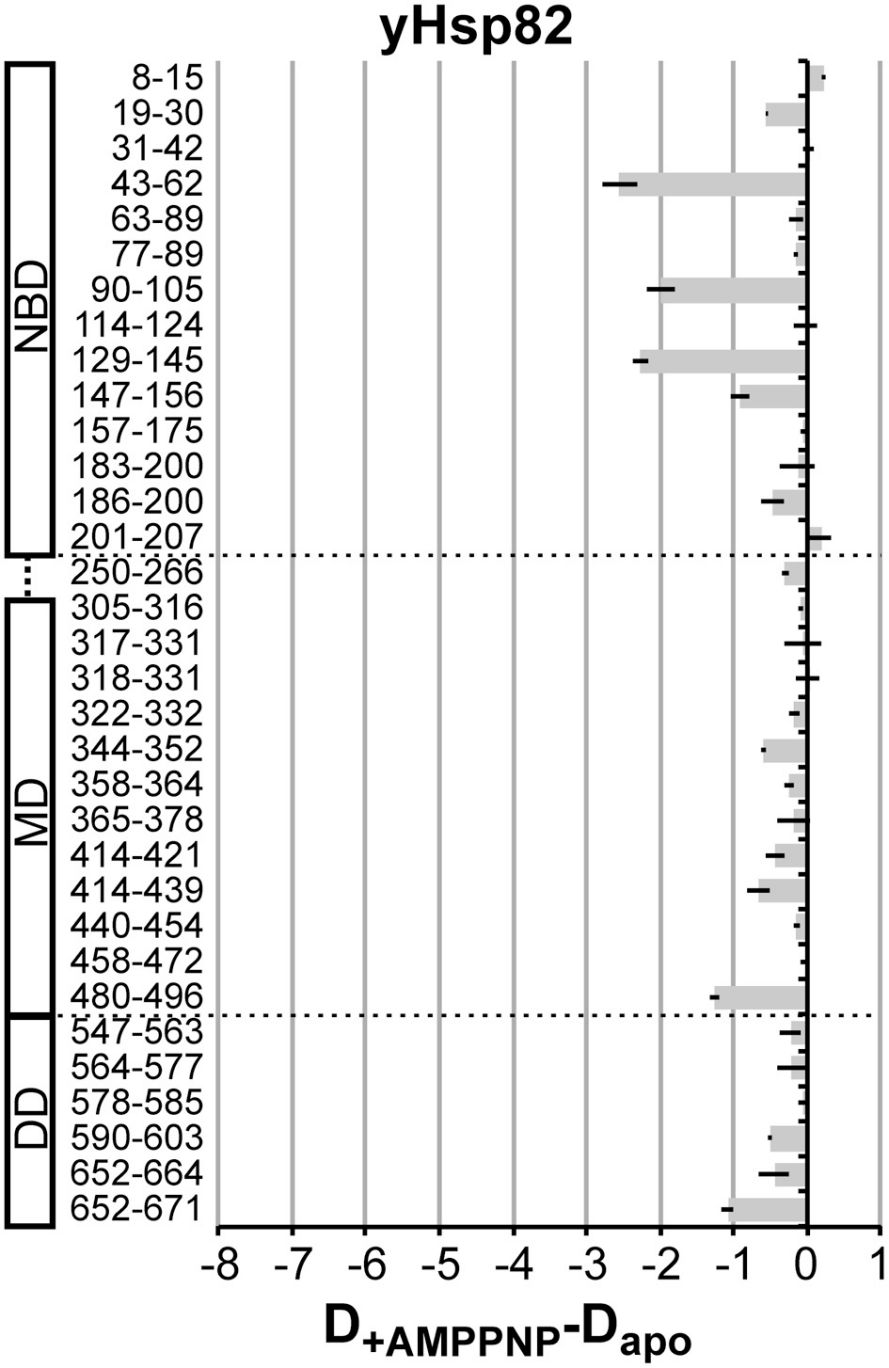
**AMPPNP-induced changes in the HX kinetics in yHsp82**. Difference plots of deuterons incorporated in the presence of AMPPNP minus deuterons incorporated in the absence of nucleotides into yHsp82 after 30 s in D_2_O. The numbers to the left indicate the corresponding peptic peptide. Presented are the means of three independent experiments. Domain structure of yHsp90 is shown left of the graph. Dashed lines indicate domain boundaries.

It was shown previously the AMPPNP leads to a more stable N-terminal dimerization in yHsp82 (Prodromou et al., 2000). We therefore repeated the experiment with yHsp82 in the presence AMPPNP (Figure 5). The observed protection in the N-terminal domain was more prominent as in the presence of ATP or ADP but again not comparable to the extent observed in EcHtpG. Interestingly, we also observed a significant protection in the MD, segment 480–496, which forms the interface to the DD, and in the dimerization interface. The protection in segment 480–496 suggests less movements of the MD relative to the DD, consistent with a more stable N-terminal dimerization. The protection in the dimer interface suggests a decrease of protomer dissociation of the Hsp90 dimer.

Taken together, significant nucleotide-dependent changes of the conformational dynamics were observed in the eukaryotic Hsp90s not only in the NBD but also in MD and DD, but the extent of protection was smaller than in the previously studied EcHtpG and not observed in all the same regions (see Figure 4C right panel for comparison, reproduced from (Graf et al., 2009); please, note the different scales).

### Subunit exchange in Hsp90 proteins

**Figure 6:**
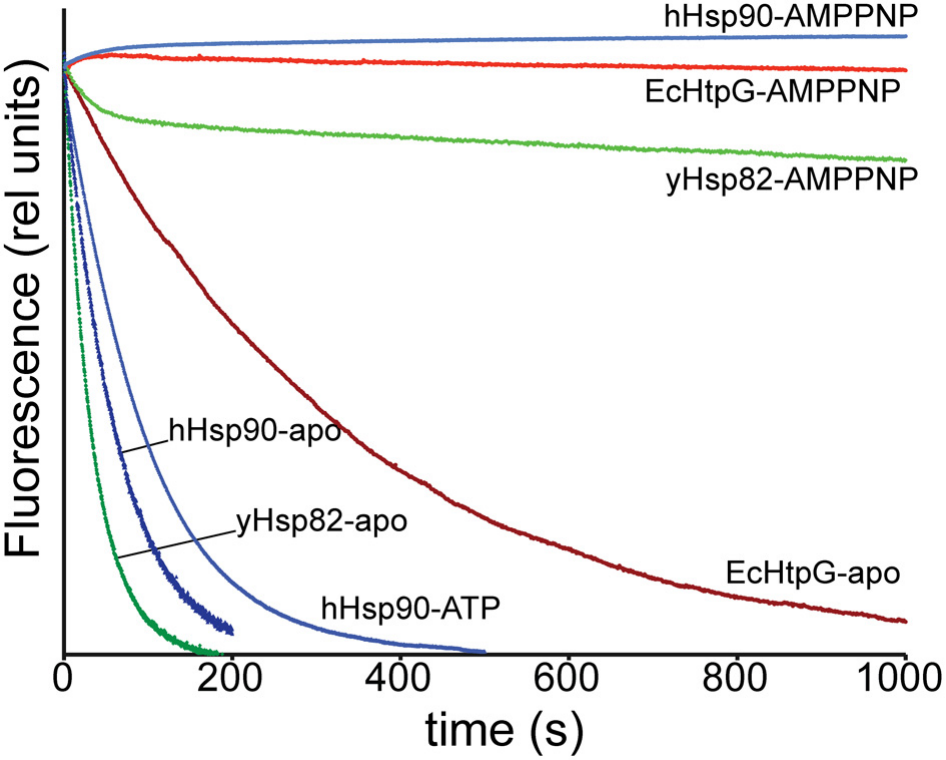
**Subunit-exchange rates differ significantly in Hsp90 proteins**. Complexes of ATTO 488 and ATTO 550 labeled single cysteine variants of EcHtpG (red), yHsp82 (green) and hHsp90 (blue) were rapidly mixed with a 10-fold excess of unlabeled protein in the absence of nucleotide (dark colors) or the presence of ATP or AMPPNP as indicated.

Our HX-MS experiments demonstrated that Hsp90 proteins exhibit striking differences in dynamics of the C-terminal helices, which are involved in the dimerization interface. These findings suggest specific differences in the dimer-monomer equilibrium and/or C-terminal opening. Therefore, we measured the subunit exchange rates using a Förster resonance energy transfer (FRET) assay as described by (Hessling et al., 2009) by labeling single cysteine variants of EcHtpG (HtpG-E58C), yHsp82 (Hsp82-E57C) and hHsp90 (Hsp90β-E20C,C366A,C412T,C521A,C564T,C589A,C590A) with ATTO488 and ATTO550. Consistent with our HX-MS data, subunit exchange rates for human and yeast Hsp90 were about 5 and 10-fold higher than for EcHtpG (Figure 6 and Table 1). ATP and ADP had no significant influence on the exchange rates for yHsp82 and EcHtpG. AMPPNP drastically reduced subunit exchange rates for both Hsp90 proteins as had been shown before for yHsp82 (Hessling et al., 2009). For human Hsp90, ATP reduced the subunit exchange rates by 30%, while ADP had no influence on the exchange rates. These data are consistent with the differences in protection observed in the segments covering the dimerization interface in EcHtpG, yHsp82 and hHsp90.

**Table 1:** **Subunit exchange rates of Hsp90 proteins**.

**Exchange rate (s^−1^)**	**apo**	**+ATP**	**+ADP**	**+AMPPNP**
HtpG	0.0028	0.0035	0.0036	≈2.8·10^−5^
Hsp82	0.030	0.027	0.030	0.034/4.8·10^−4^
Hsp90β	0.016	0.010	0.016	0

### Changes in conformational dynamics of yHsp82 upon binding of Cpr6

Since the eukaryotic Hsp90s are regulated by a number of co-chaperones, which are absent in *E. coli*, we hypothesized that the eukaryotic proteins may need the help of co-chaperones to arrive at the same tensed state as EcHtpG. To address this hypothesis, we analyzed the conformational changes of yHsp82 in the presence of the co-chaperones Cpr6, Sti1 and Sba1.

**Figure 7:**
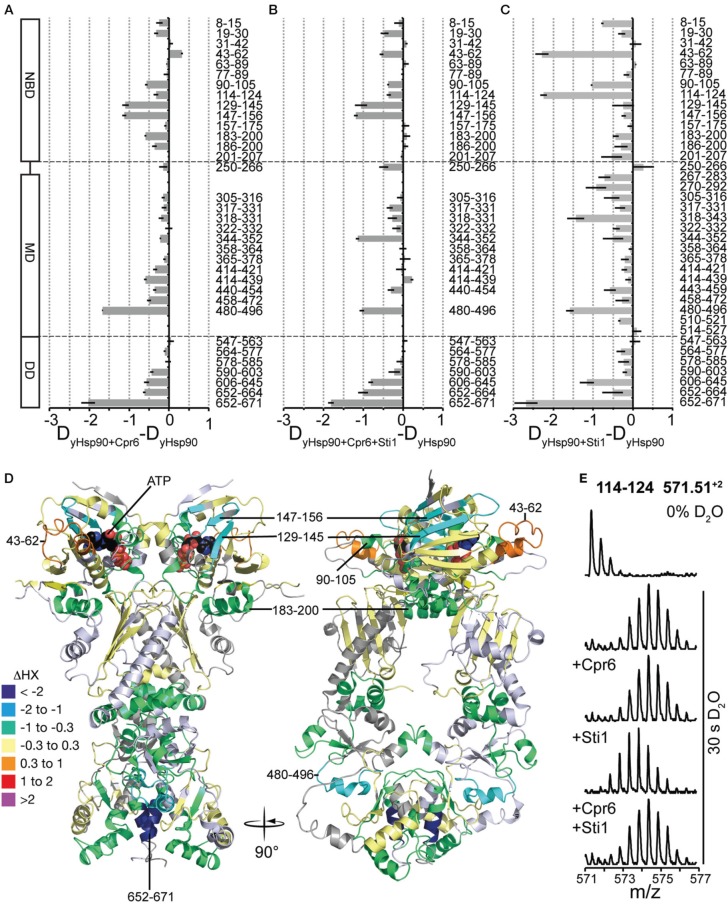
**Effects of Cpr6 and Sti1 on the conformational dynamics of yHsp82. (A)** Difference plots of deuterons incorporated into yHsp82 in the presence of Cpr6 minus deuteron incorporation in the absence of Cpr6 after 30 s HX. The numbers to the right indicate the corresponding peptic peptides, the domain structure is shown to the left. Error bars represent standard error of three independent experiments. **(B)** Difference plots of deuterons incorporated into yHsp82 in the presence of Cpr6 and Sti1 minus deuteron incorporation in the absence of Cpr6 and Sti1 after 30 s HX. **(C)** Difference plots of deuterons incorporated into yHsp82 in the presence of Sti1 minus deuteron incorporation in the absence of Sti1 after 30s HX (data from Lee et al., 2012). **(D)** Cartoon representation of yHsp82 (PDB ID 2CG9) colored according to difference of deuteron incorporation into yHsp82 in the presence of Cpr6 minus deuteron incorporation into yHsp82 in the absence of Cpr6 as indicated in the color scheme (= heat map for data shown in **A**). **(E)** Original spectra of the peptic peptide amino acids 114–124 of yHsp82 (m/z 571.51, *z* = 2) before HX (0% D_2_O), or after 30 s in D_2_O in the absence of co-chaperones, or in the presence of Cpr6, Sti1 or both Cpr6 and Sti1, from top to bottom as indicated.

Cpr6 is a yeast member of the family of large peptidyl-prolyl-cis/trans-isomerases, which interacts with the C-terminal MEEVD-motif of Hsp90 through its tetratricopeptide repeat (TPR) domain. When performing HX-MS experiments with yHsp82 in the presence of Cpr6 we could not observe the interaction of Cpr6's TPR-domain and yHsp82's MEEVD-motif directly, because the C-terminal 38 residues were not covered by peptic peptides that we could detect consistently in HX-MS experiments. However, we detected significant protection in all three domains of yHsp82 (Figures 7A,D). Most prominent was the protection in helix α18 (aa 665–671), which is involved in the dimer interface, suggesting that Cpr6 stabilizes C-terminal dimerization. Protection was also observed in segment 480–496, which is situated at the hinge between MD and DD, suggesting reduced relative rotational movement between MD and DD. Some protection was also observed in the NBD notably in the β-sheet (aa 129–156), in the ATP lid (aa 90–105) and in helix α5 (aa 183–200), which is at the interface between NBD and MD in the crystal structure of the closed conformation of yHsp82 (Ali et al., 2006), suggesting that the relative movement of NBD and MD is also reduced in the presence of Cpr6. Of note, only a small protection was observed in regions in the NBD (aa 8–30) that interact with each other during N-terminal dimerization, suggesting that Cpr6 does not significantly stabilize the N-terminally dimerized state of Hsp90.

**Figure 8:**
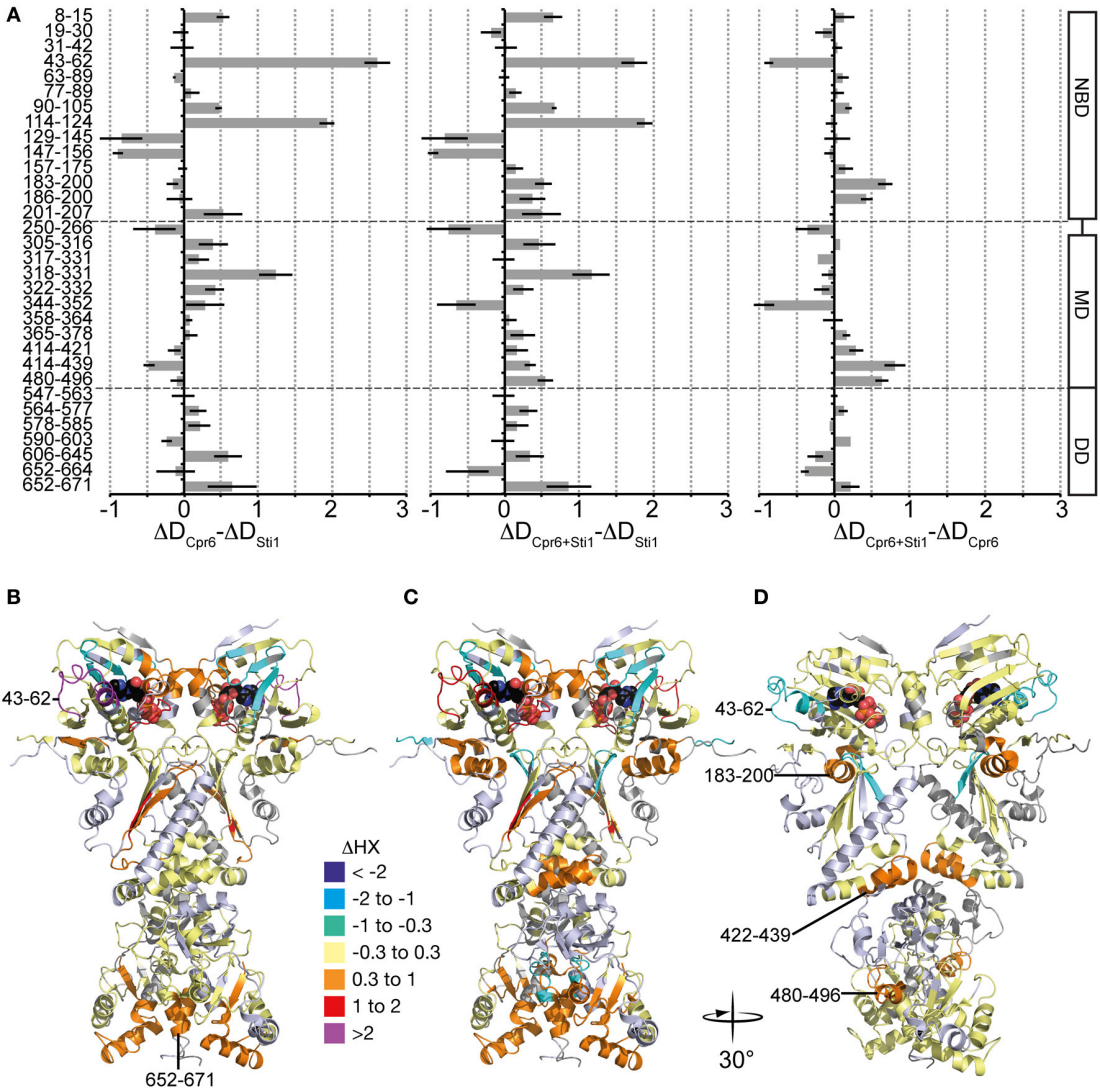
**Comparison of Cpr6 and Sti1-induced changes in dynamics of yHsp82. (A)** left panel, Difference of Cpr6-induced changes in HX in yHsp82 as compared to Sti1-induced changes [data (Figure 7A) -data (Figure 7C)]; middle panel, Difference in changes in HX of yHsp82 induced by simultaneous presence of Cpr6 and Sti1 as compared to presence of Sti1 alone [data (Figure 7B) -data (Figure 7C)]; right panel, Difference in changes in HX of yHsp82 induced by simultaneous presence of Cpr6 and Sti1 as compared to presence of Cpr6 alone [data (Figure 7B) -data (Figure 7A)]. **(B–D)** Cartoon representations of yHsp82 (PDB ID 2CG9) colored according to the difference plots shown in **(A)**, left panel **(B)**, middle panel **(C)**, and right panel **(D)** as indicated.

This exchange pattern of yHsp82 in the presence of Cpr6 has similarities but also striking differences to the pattern previously observed for yHsp82 in the presence of Sti1 (see Figure 7C, data from Lee et al., 2012 for comparison), which becomes more obvious when presented as difference plot (Figure 8A, left panel and Figure 8B, heat map). Positive values indicate regions that are more protected in the presence of Sti1 than in the presence of Cpr6, negative values are more protected in the presence of Cpr6 than in the presence of Sti1. In the presence of Sti1 segments 43–62, 114–124, and 318–331 were more protected than in the presence of Cpr6. In contrast, region 129–156 was more protected in the presence of Cpr6 than in the presence of Sti1.

Kinetic data demonstrated that Cpr6 can bind at the same time as Sti1 and that Cpr6 promotes dissociation of Sti1 in the function cycle of Hsp90 (Li et al., 2010). To understand the molecular basis of this observation we determined deuteron incorporation in the presence of stoichiometric concentrations of Cpr6 and Sti1 (yHsp82:Cpr6:Sti1 = 2:1:1; Figure 7B). Overall, the observed pattern is more similar to the pattern induced by Cpr6 than the Sti1-induced pattern (Figures 8A,C,D). Notably, the Sti1-induced protection in regions 43–62 and 114–124 are strongly reduced as compared to the situation in the presence of Sti1. In two regions (aa 250–266, 344–352) protection was observed when both co-chaperones were present, while no significant protection could be detected in the presence of either co-chaperone alone.

Taken together, our data demonstrate that Cpr6 and Sti1 induce different protection pattern in yHsp82 indicating that they stabilize yHsp82 in defined distinguishable conformations.

### Changes in conformational dynamics of yHsp82 upon binding of Sba1

**Figure 9:**
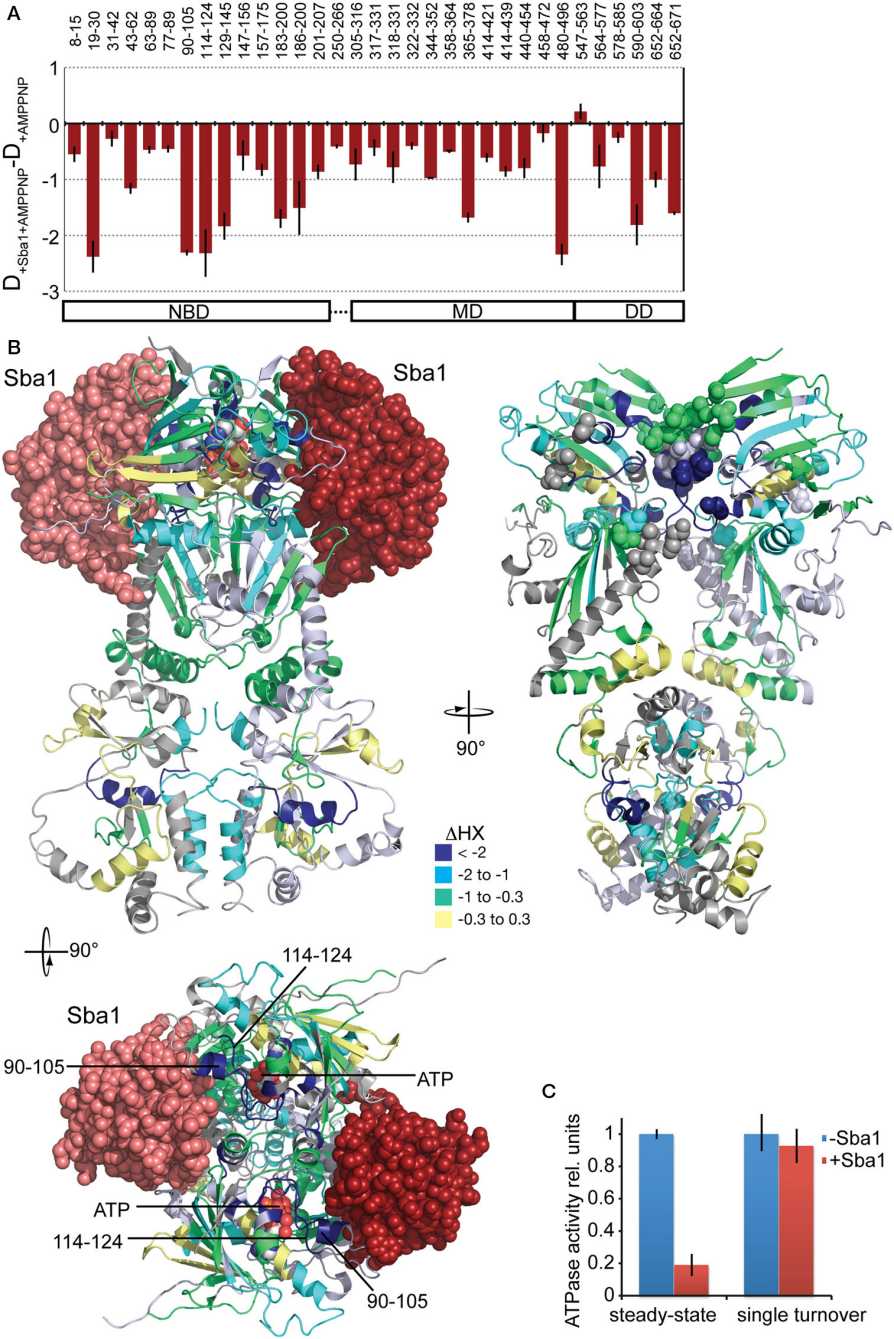
**Effects of Sba1 and AMPPNP on the conformational dynamics of yHsp82. (A)** Difference plots of deuterons incorporated into yHsp82 in the presence of Sba1 and AMPPNP minus deuteron incorporation in the absence of Sba1 but presence of AMPPNP after 30 s HX. The numbers on top indicate the corresponding peptic peptides, the domain structure is shown on the bottom. Error bars represent standard error of three independent experiments. **(B)** Left panel, crystal structure of yHsp82Δ(221–255)-A107N (cartoon representation) in complex with Sba1 (spheres) and AMPPNP. Right panel, same structure rotated as indicated without Sba1 and with Sba1-interacting residues shown as spheres. yHsp82 is colored according to the difference between deuteron incorporation in the presence of Sba1 and deuteron incorporation in the absence of Sba1. Lower panel is a top view of the upper left panel. Segments of the ATP-lid observed in HX experiments are indicated. **(C)** Sba1 does not inhibit the ATPase activity of yHsp82 under single-turnover conditions. ATPase activity of yHsp82 was determined under steady-state (2 μM Hsp82, 3 mM ATP) and single turnover (50 μM yHsp82, 40 μM ATP) conditions in the absence and presence of Sba1 (60 μM).

The co-chaperone Sba1/p23 is known to form a complex with Hsp90 in the presence of AMPPNP (Johnson and Toft, 1995; Sullivan et al., 1997; Fang et al., 1998; Prodromou et al., 2000; Richter et al., 2004; Siligardi et al., 2004). We measured HX in yHsp82 in the presence of Sba1 and AMPPNP and compared the results with HX in yHsp82 in the presence of AMPPNP. The difference plot in Figure 9A shows that a protection is observed in the entire protein. The crystal structure of yHsp82Δ(221–255)-A107N variant in complex with Sba1 and AMPPNP revealed residues 12–21, 27, 108–113 (ATP lid), 151–155, 315, 375, 387, 388, and 391 of Hsp82 as major interaction sites for Sba1 (Figure 9B). For most part our data are consistent with these interaction sites since we observed a significant protection in segments that contain the respective residues (segments 8–15, 19–30, 147–156, 365–378) or that are immediately adjacent to the interacting residues (90–105, 114–124) (Figures 9A,B). In addition, we found many segments in all three domains that exhibit strong protection in the presence of Sba1 but that do not have any direct contact sites. In particular, strong protection was observed in residues 365–378, which in the crystal structure (Ali et al., 2006) make polar and hydrophobic contacts to residues 114–120 in the NBD; in the dimer interface (segment 652–671), indicating reduced dissociation of the yHsp82 dimer and/or C-terminal opening; and in the hinge region between MD and DD (segment 480–496), suggesting reduced movement of the MD relative to the DD. These data are consistent with the stabilization of yHsp82 in the closed conformation as suggested by biochemical experiments and revealed in the crystal structure (Richter et al., 2004; Ali et al., 2006). Our data demonstrate that Sba1 reduces the conformational dynamics of the ATP lid as indicated by the protection in residues 90–105 and 114–124, which is consistent with mutant analysis (Siligardi et al., 2004). This suggests that Sba1 inhibits ATP hydrolysis by preventing product release. To test this hypothesis we performed single-turnover ATPase assays in the absence and presence of Sba1, since product release is not relevant in this assay. As shown in Figure 9C Sba1 did not inhibit significantly the ATPase activity of yHsp82 under single turnover conditions, which contrasts the observed inhibition under steady state conditions, demonstrating that Sba1 inhibits product release. Thus, Sba1 prolongs the life-time of the closed conformation of Hsp90 as previously proposed (Ali et al., 2006; Prodromou, 2012) and consistent with Sba1's proposed ability to stabilize Hsp90 client complexes (Richter et al., 2004; Siligardi et al., 2004; McLaughlin et al., 2006).

## Discussion

Our analysis of the conformational dynamics of eukaryotic Hsp90 proteins yielded several insights into structure and functioning of Hsp90 proteins. (1) The conformational dynamics of the eukaryotic Hsp90s is much higher than the dynamics of previously studied EcHtpG. (2) As observed for EcHtpG, nucleotide-induced conformational changes are not restricted to the NBD but observed throughout the Hsp90 proteins consistent with published data (Phillips et al., 2007). (3) Eukaryotic Hsp90s are almost as dynamic in the presence of nucleotides as in the apo state consistent with single molecule data (Mickler et al., 2009), and ATP or AMPPNP do not induce a state similar to the ATP-bound tensed state of EcHtpG. (4) Co-chaperones stabilize Hsp90 in distinct conformations. (5) Co-chaperones influence each others binding by altering the conformation of Hsp90. (6) Sba1 stabilizes Hsp90 in the hydrolysis competent conformation but inhibits ATP hydrolysis by slowing down product release.

Consistent with single molecule measurements on yHsp82 and EcHtpG (Mickler et al., 2009; Ratzke et al., 2012), our data provide strong evidence for the hypothesis that the eukaryotic Hsp90 proteins are probabilistic machines with many different conformational states that are separated only by small energy barriers, while EcHtpG is a deterministic machine with a defined reaction cycle driven by ATP binding and hydrolysis. For eukaryotic Hsp90s, nucleotides alone do not drive a defined reaction cycle, nucleotides and co-chaperones are necessary for an ordered progression through the ATPase cycle.

The higher conformational dynamics in the eukaryotic Hsp90 proteins as compared to the EcHtpG could have several different reasons. The eukaryotic proteins evolved to bind a large number of natively folded proteins, many of which are unrelated in sequence or structure [http://www.picard.ch/downloads/Hsp90interactors.pdf (Picard, 2006)]. Such a large spectrum of clients may rely on a highly flexible structure to allow Hsp90 to quasi mold into different shapes to provide an optimal interface for client binding. Although it is currently unknown how many and what kind of clients are bound by EcHtpG, it is assumed that these are much lower in number and either not essential themselves or not stringently dependent on EcHtpG, since EcHtpG is not essential under any conditions tested so far and *htpG* deletion has a very mild temperature sensitivity phenotype at the upper temperature limit for *E. coli* growth. Alternatively or in addition, the dynamics of the eukaryotic proteins could also be linked to the interaction with the plethora of co-chaperones. No co-chaperones were identified for EcHtpG so far. While EcHtpG appears to work as a stand-alone machine, the eukaryotic Hsp90s may require the co-chaperones not only to regulate progression through the ATPase cycle but also to stabilize certain parts of the eukaryotic Hsp90 proteins as required for the binding of specific clients.

Consistent with both hypotheses is the fact that the differences in solvent accessibility between EcHtpG and the eukaryotic Hsp90 mainly reside in the MD and the DD, both of which are implicated in client and co-chaperone binding (Sato et al., 2000; Meyer et al., 2003; Harris et al., 2004). The two co-chaperones tested in this study, Cpr6 and Sba1, both stabilized parts in MD and DD of yHsp82.

HX-MS suggests that the ATPase cycle of all Hsp90s involve similar conformational changes. ATP-induced protection in human and yeast Hsp90s was particularly prominent in the segments, which constitute the hinges of the ATP-lid (residues 99–114 and 126–133 in hHsp90, 90–105 and 114–124 in yHsp82 and 89–98 and 120–127 in EcHtpG). These nucleotide-dependent changes are consistent with structural studies since these segments are found in different conformations in crystal structures of the apo NBD of Hsp90s [PDB ID code 1AH6, 1AH8, 1YES; (Prodromou et al., 1997a,b; Stebbins et al., 1997)] as compared to the full-length structures of yHsp82 and EcHtpG in complex with AMPPNP and Sba1/p23 or ADP [PDB ID codes 2CG9 and 2IOP; (Ali et al., 2006; Shiau et al., 2006)]. They are also consistent with an NMR study of the isolated NBD (Dehner et al., 2003) and of full-length human Hsp90 (Karagöz et al., 2011). However in contrast to the NMR study of full-length Hsp90 (Karagöz et al., 2011), we did not observe any indications of asymmetry in ATP bound Hsp90. The reason for this might be that the observed changes in HX are rather small, making it difficult to detect bimodal distributions, the hallmark of coexisting populations with difference in conformation. Also, the interconversion between the two conformations might be fast on the timescale of our experiments, which would prevent detection by HX-MS.

In addition to the stabilization in the ATP-lid, we found an ATP-induced stabilization in segment 394–415 in hHsp90, a similar protection we observed previously in 318–334 in EcHtpG (Graf et al., 2009). The changes in solvent accessibility in this segment supports the currently favored mechanism of ATP hydrolysis, which assigns a crucial role to arginine 380 in yHsp82 (392 in hHsp90, 336 in EcHtpG) (Meyer et al., 2003, 2004; Ali et al., 2006). A contact between nucleotide and arginine 380 was shown in the crystal structure of full-length yHsp82 in complex with AMPPNP and Sba1/p23 (Ali et al., 2006). Our data now demonstrate that a communication between nucleotide and this arginine-containing loop also occurs in hHsp90 in the presence of ATP and the absence of the Sba1/p23 co-chaperone as previously shown for EcHtpG (Graf et al., 2009). Our data seem to contrast NMR data on full-length human Hsp90 that did not detect perturbations in the chemical shift of the δ-methyl groups of Ile in the MD (Karagöz et al., 2011). The difference between our HX-MS experiments and NMR techniques are the timescale of incubation and data collection, respectively. The full-length crystal structure of yHsp82 also shows an N-terminal dimerization with an exchange of the first β-strand. In the structure this strand exchange is stabilized by 4 hydrogen bonds. The stabilization observed in the first segments of hHsp90 and yHsp82 was only very small, presumably because any N-terminal dimerization is very transient in the presence of ATP and the absence of stabilizing mutations (Ala107Asn; linker deletion) and co-chaperones like Sba1/p23 like in the crystal structure (Ali et al., 2006). In contrast, in EcHtpG we observed a very prominent protection of almost 8 amide hydrogens in this segment. This observation would be consistent with a stable N-terminal dimerization with strand exchange. The larger number of hydrogen bonds formed in EcHtpG as compared to yHsp82 could be necessary for lack of stabilizing co-chaperones.

Our HX-MS data of yeast and human Hsp90 in the presence of ADP does not provide any evidence for a closed conformation as previously suggested based on electron microscopic data (Shiau et al., 2006; Southworth and Agard, 2008).

Comparison of HX pattern induced by Cpr6 and Sti1 revealed characteristic differences. Most striking is the segment 43–62, which is strongly protected in the presence of Sti1 but slightly deprotected in the presence of Cpr6 (Figures 7A,C). This region was suggested to be a direct interaction site between Sti1 and yHsp82 since it could be cross-linked by a cross-linker positioned in this region (Lee et al., 2012). Cpr6 does not seem to interact with this region. Similarly, the ATP lid (aa 90–105 and aa 114–124) in the NBD and part of the β-sheet of the MD (aa 318–331) are more protected in the presence of Sti1 than in the presence of Cpr6. In contrast, parts of the β-sheet of the NBD (aa 129–156) are more protected in the presence of Cpr6 than in the presence of Sti1.

When adding Sti1 and Cpr6 to yHsp82 at the same time, one of our major concerns was that we may have yHsp82_2_-Sti1_2_ and yHsp82_2_-Cpr6_2_ complexes in addition to the envisioned yHsp82_2_-Sti1-Cpr6 complex. However, the former two complexes would cause a bimodal distribution in the isotope distribution of peptic fragments that differ between the two complexes (e.g. 43–62 and 114–124). Even in a 1:2:1 mixture of yHsp82_2_-Sti1_2_: yHsp82_2_-Sti1-Cpr6: yHsp82_2_-Cpr6_2_ complexes this should have been visible in a broadening of the isotope distribution. However, this was not observed (see Figure 7E). These data are consistent with published FRET data (Li et al., 2010). The observation that the Sti1-induced protection in region 43–64 is strongly reduced in the simultaneous presence of Cpr6 suggests that Cpr6 destabilizes the interaction of Sti1 with the NBD. The protection in the β-sheet of the NBD (aa 129–156) in the presence of Sti1 and Cpr6 are as high as in the presence of Cpr6 alone, suggesting that the conformational dynamics of the NBD are dominated by the interaction with Cpr6. Also in the presence of Sti1 and Cpr6 the protection of the β-sheet of the MD (aa 318–331) are reduced as compared to the presence of Sti1 alone, which might indicate that interaction of Sti1 with this region is reduced. Surprisingly, we did not observe a bimodal distribution in the regions where the protection pattern differs between Sti1 and Cpr6 (see Figure 7E). This would be expected, if Sti1 stably interacts with one protomer and Cpr6 with the other. As no bimodal distributions are observed, both yHsp82 protomers are identical within the time scale of our experiments (30 s). There are two possible explanations for this observation. Both co-chaperones might influence both protomers alike irrespective to which protomer they are bound. Alternatively, the interaction of Sti1 and Cpr6 with yHsp82 might be dynamic allowing frequent switching of the protomer to which they are bound. The long flexible C-terminal linker that connects the MEEVD motif to the C-terminal dimerization domain and that we previously dubbed fishing line with the MEEVD-motif as hook might allow protomer switching without dissociation from the Hsp90. Both models are consistent with the fact that Sti1 inhibits the ATPase activity of both protomers even at a stoichiometry of 1:2 Sti1:yHsp82 (Li et al., 2010).

Taken together, our data show that although the primary interaction site for Sti1 and Cpr6 is the MEEVD motif, both cause stabilization of regions in all three domains of yHsp82 that differs significantly between the two co-chaperones. Our data further suggest that Cpr6 promotes dissociation of Sti1 by destabilizing its contacts to a loop in the NBD and a β-sheet in the MD.

Sba1 binding in the presence of AMPPNP induced a stabilization of yHsp82 in our HX-MS experiments. This was not unexpected since Sba1 and its mammalian homolog p23 were shown to stabilize the closed state of Hsp90 (Johnson and Toft, 1995; Johnson et al., 1996; Sullivan et al., 1997; Prodromou et al., 2000). However, with an average of 2 protected amide protons for the 5 most protected peptic fragments the magnitude of stabilization in the N-terminal domain was significantly lower than the stabilization induced by ATP or AMPPNP in EcHtpG [>5 H for the 5 most protected peptic fragments; Figure 4C; (Graf et al., 2009)]. The most likely explanation for this difference is that the interaction of Sba1/p23 is dynamic and association-dissociation cycles happen during the 30-s-incubation in D_2_O. This is consistent with cross-linking data (C-T. L. and M.P.M, unpublished results) and the fact that for crystallization of full-length yHsp82 in complex with Sba1/p23 the linker between NBD and MD was shortened by 34 residues (221–255) and, in addition, Ala107 replaced by Asn, an amino acid replacement known to stabilize the closed conformation and to increase the ATPase activity. The protection in the NBD is consistent with a previous NMR study (Karagöz et al., 2011). The protected segments 19–30 and 114–124 include residues corresponding to Ile27, Ile28 and Ile122 in human Hsp90β (19, 20 and 114 yHsp82 numbering) where chemical shifts changes were detected in the presence of p23.

We also observed protection in the MD and DD, in particular in the dimerization interface. Some of the protected areas in the MD (e.g., 365–378) are consistent with the known crystal structure (Ali et al., 2006), caused either by direct binding of Sba1 to yHsp82 or by contacts between MD and NBD in the closed conformation. However, there are also parts of the MD for which protection cannot be explained in this way. In the NMR study some isoleucine residues in the MD also exhibited a shift perturbation (Karagöz et al., 2011). There are two alternative, mutually non-exclusive explanations for these observations. In the crystal structure of yHsp82 in complex with Sba1 the C-terminal 81 residues of Sba1 (136–216) were not present since they are unstructured (Weikl et al., 1999). Interaction of these residues with the MD could be responsible for the observed protection in our experiments. Alternatively, stabilization of the N-terminally dimerized conformation of yHsp82 may reduce the dynamics of the MD. The protection observed in the dimerization interface suggests that the slow subunit exchange in the presence of AMPPNP is further reduced upon binding of Sba1.

Several laboratories published earlier that Sba1/p23 inhibits the ATPase activity of Hsp90 (Panaretou et al., 2002; Richter et al., 2004; Siligardi et al., 2004; McLaughlin et al., 2006). Steady-state kinetics suggested a mixed mechanism of inhibition for human p23 (McLaughlin et al., 2006). Interestingly, the degree of inhibition by yeast Sba1 seemed to level off at 50% residual ATPase activity. Based on these data and on earlier observations that Sba1/p23 stabilizes the Hsp90-client complex and that hydrolysis of ATP by Hsp90 leads to client release, it was proposed that Sba1/p23 stabilizes the pre-hydrolysis closed conformation of Hsp90 (Young and Hartl, 2000; Richter et al., 2004; Siligardi et al., 2004; McLaughlin et al., 2006; Prodromou, 2012). The crystal structure of yHsp82 with Sba1 shows a hydrolysis competent conformation, but could not distinguish whether Sba1 inhibits ATP hydrolysis by stabilizing the pre-hydrolysis or post-hydrolysis state (Ali et al., 2006). In addition, it was shown recently that the cochaperones Aha1, which stimulates the ATPase activity of Hsp90 by promoting the closed conformation appears to act before Sba1/p23 in the ATPase cycle of Hsp90 (Li et al., 2013). Our HX-MS experiments indicate that Sba1 stabilizes the ATP binding lid and most likely the N-terminally dimerized conformation by interacting with both NBDs of the Hsp90 dimer. Our single-turnover experiments demonstrate that Sba1 does not inhibit the hydrolysis step itself but the post-hydrolysis product release and most likely the dissociation of the docked NBDs. Whether Sba1 enters the Hsp90 complex before hydrolysis or after hydrolysis before the reopening of the closed conformation cannot be decided on the basis of our or previous data. Sba1 might bind with its C-terminal unstructured tail to the Hsp90-bound client waiting for the Aha1-induced N-terminal dimerization of Hsp90 and then bind with high association rates to the docked NBDs. The consequence of this interaction is most likely a stabilization of the Hsp90-client complex as has been proposed earlier (Kosano et al., 1998; Morishima et al., 2003; Ali et al., 2006; McLaughlin et al., 2006).

In summary, our data demonstrate that the eukaryotic Hsp90s have increased conformational dynamics as compared to EcHtpG and do not react with large changes in conformational dynamics to binding of nucleotides. In contrast, they need cochaperones to be stabilized in defined conformations necessary for binding and activation of the plethora of their clients. It might be a general tendency in protein evolution within the eukaryotic lineages to lose rigidity and deterministic behavior in order to gain versatility of interaction partners and regulatory potential for cofactors.

## Materials and methods

### Reagents

Fine chemicals were purchased from SIGMA-Aldrich (St. Louis, MO), ATP and ADP were obtained from Roche Applied Science, Mannheim, Germany. Radicicol (RA) was obtained from IRIS Biotech GmbH, Marktredwitz, Germany. Deuterium oxide was purchased from Euriso-top, Gif-sur-Yvette, France. His-tagged Ulp1 was prepared in-house.

### Protein expression and purification

Human Hsp90β, yeast Hsp82, yeast Hsc82, Cpr6, and Sba1 were cloned into the bacterial expression vector pCA528 encoding an N-terminal His_6_-Smt3 tag (Andréasson et al., 2008). The fusion proteins were overexpressed in the *E. coli* strain BL21(DE3)Star/pCodonPlus (Invitrogen). The cultures were grown to OD_600_ = 0.6 and expression was induced with 0.5 mM IPTG for 5 h at 30°C. Cells were lysed by a microfluidizer (Avestin EmulsiFlex-C5) in lysis buffer A (20 mM HEPES/KOH pH 7.5, 100 mM KCl, 5 mM MgCl_2_, 10% glycerol, 4 mM β-mercaptoethanol) and 5 mM PMSF, 1 mM Pepstatin A, 1 mM Leupeptin, 1 mM Aprotinin. The lysate was clarified by centrifugation (40,000 rpm for 30 min) and incubated with Ni-IDA-matrix (Protino, Macherey-Nagel) for 30 min. After incubation, the matrix was washed with buffer A and bound protein eluted with buffer A containing 250 mM imidazole. The eluted fusion proteins were supplemented with Ulp1 protease, which cleaved the His_6_-Smt3 tag and the mixture was dialyzed overnight against buffer A containing 10 mM KCl. Cleaved recombinant proteins were recovered in the flow-through fractions after a second incubation with Ni-IDA matrix whereas the N-terminal His_6_-Smt3 tag and Ulp1 remained on the column. Proteins were further purified by anion exchange chromatography (ReSourceQ, GE Healthcare) with a linear gradient of 0.01–1 M KCl), followed by Superdex 200 size-exclusion chromatography in buffer B (20 mM HEPES 7.6, 300 mM KCl, 5% glycerol, 1mM DTT) and finally dialyzed against storage buffer (40 mM HEPES, pH 7.5, 50 mM KCl, 5 mM MgCl_2_, 10% glycerol, 4 mM β-mercaptoethanol). The purity and molecular mass was verified by SDS-PAGE and HPLC-electrospray ionization mass spectrometry, confirming the correct primary sequence containing only the N-terminal start-methionine. The purified Hsp90 proteins were checked to be nucleotide-free by anion-exchange chromatography (ReSourceQ) and UV detection by 254 nm.

### Hydrogen-exchange experiments, mass spectrometry and data processing

Nucleotide-free Hsp90 proteins (40 μM) were pre-incubated with a large excess of buffered ATP (120 mM), ADP (60 mM), or AMPPNP (60 mM) for 10 min at 30°C to ensure complete binding. In all cases the ligand occupancy of Hsp90 as calculated from the published dissociation equilibrium constants using the quadratic solution of the binding equilibrium was more than 99% during pre-incubation and more than 95% during incubation in D_2_O.

HX experiments, mass spectrometry analysis and data processing were performed as described earlier (Rist et al., 2003, 2006; Graf et al., 2009). All experiments were performed at least 3 times independently.

### Fluorescence measurement

For the determination of subunit exchange EcHtpG-E58C, yHsp82-E57C and hHsp90β-E20C,C366A,C412T,C521A,C564T,C589A,C590A were labeled with ATTO 488 maleimide and ATTO 550 maleimide (ATTO-TEC GmbH). Corresponding ATTO 488 and ATTO 550 labeled proteins were combined (400 nM each) and rapidly mixed 1:1 with unlabeled protein (20 μM) in a stopped-flow device (Applied Photophysics) with 480 nm excitation and 590 nm cut-off filter.

### ATPase assays

Steady-state and single-turnover ATPase assays were determined as described earlier (Ali et al., 1993; Graf et al., 2009) except that 25 mM HEPES-KOH pH 7.5, 20 mM KCl, 5 mM MgCl_2_ was used as buffer.

## Author contributions

Christian Graf cloned genes, constructed and purified wild type and mutant proteins; performed experiments and analyzed data for Figures 1–4; prepared Figures 1, 2A; contributed to manuscript writing. Chung-Tien Lee constructed and purified wild type and mutant proteins; performed experiments and analyzed data for Figures 2C, 3, 4B,C, 5, 6C, 8; contributed to manuscript writing. L. Eva Meier-Andrejszki purified Cpr6 and performed experiments and analyzed data for Figures 7A,B; Minh T. N. Nguyen constructed mutant variants and performed ATPase assays (Figure 9C). Matthias P. Mayer designed and supervised experiments, analyzed data, prepared figures and wrote the manuscript.

### Conflict of interest statement

The authors declare that the research was conducted in the absence of any commercial or financial relationships that could be construed as a potential conflict of interest.
